# Transesterification with CE15 glucuronoyl esterase from *Cerrena unicolor* reveals substrate preferences

**DOI:** 10.1007/s10529-023-03456-x

**Published:** 2023-12-27

**Authors:** Valentina Perna, Jane Wittrup Agger

**Affiliations:** Department of Biotechnology and Biomedicine, Technical University of Denmark, 2800 Kgs Lyngby, Denmark

**Keywords:** Glucuronoyl esterase, Lignin-carbohydrate complexes, Substrate specificity, Transesterification

## Abstract

**Purpose:**

Glucuronoyl esterases (GE, family CE15) catalyse the cleavage of ester linkages in lignin-carbohydrate complexes (LCCs), and this study demonstrate how transesterification reactions with a fungal GE from *Cerrena unicolor* (*Cu*GE) can reveal the enzyme’s preference for the alcohol-part of the ester-bond.

**Methods:**

This alcohol-preference relates to where the ester-LCCs are located on the lignin molecule, and has consequences for how the enzymes potentially interact with lignin. It is unknown exactly what the enzymes prefer; either the α-benzyl or the γ-benzyl position. By providing the enzyme with a donor substrate (the methyl ester of either glucuronate or 4-*O*-methyl-glucuronate) and either one of two acceptor molecules (benzyl alcohol or 3-phenyl-1-propanol) we demonstrate that the enzyme can perform transesterification and it serves as a method for assessing the enzyme’s alcohol preferences.

**Conclusion:**

*Cu*GE preferentially forms the γ-ester from the methyl ester of 4-*O*-methyl-glucuronate and 3-phenyl-1-propanol and the enzyme’s substrate preferences are primarily dictated by the presence of the 4-*O*-methylation on the glucuronoyl donor, and secondly on the type of alcohol.

**Supplementary Information:**

The online version contains supplementary material available at 10.1007/s10529-023-03456-x.

## Introduction

Glucuronoyl esterases cleave ester-linked lignin-carbohydrate complexes (LCCs) in lignocellulosic biomass (Mosbech et al. 2018). In this way, their catalytic action assists in decomposing the plant cell wall matrix by removing some of the covalent interpolymeric linkages that sustain lignocellulose recalcitrance.

**Fig. 1:**
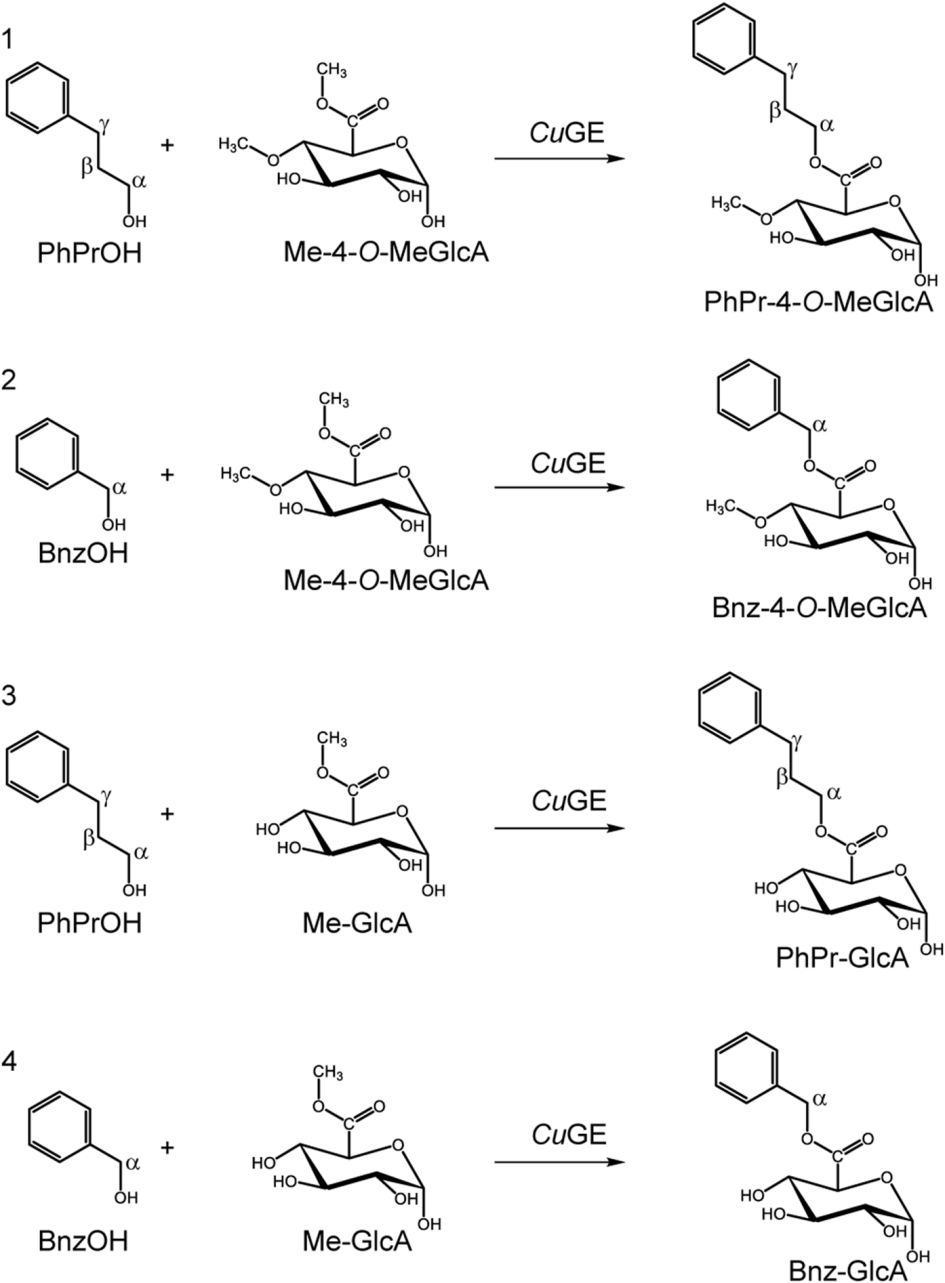
Four transesterification reactions catalyzed by *Cu*GE performed by combining either one of the two different donor substrates, methyl-4-*O*-methyl-glucuronate (1 and 2) and methyl-glucuronate (3 and 4) with either 3-phenyl-1-propanol (PhPrOH, reaction 1 and 3) or benzyl alcohol (BnzOH, reaction 2 and 4) as acceptor substrates

As literature sees it today, ester-linked LCCs exist in vivo between the α-1,2-linked-D-glucuronoyl substitutions of glucuronoxylan and the aliphatic hydroxyl groups (Balakshin et al. 2011; Yuan et al. 2011). An important additional feature observed in many wood and cereal types of biomass, is the 4-*O*-methylation on said glucuronoyl, which creates a unique plant-based trademark for these aldouronic acids. We commonly agree on the carbohydrate side of the LCC, but the exact nature of the aliphatic alcohol on lignin remains a matter of debate (Giummarella et al. 2019; Sapouna & Lawoko 2021). In the most abundant lignin-substructures, two alcohol moieties present themselves as possible esterification points, namely the α- and the γ-positioned ones (Fig. 1, structure 1 vs. structure 2), and whichever is most dominant in vivo is difficult to verify, because the occurrence is low, and because they prove difficult to isolate and annotate exactly. Ester bound acids tend to migrate (Puchart et al. 2020) and extensive sample handling in order to enrich these linkages may introduce artifacts. Hence, de novo ester synthesis in the plant cell may start in one position and migrate to a different one, as the cell wall matures (Li & Helm 1995).

Glucuronoyl esterases of both fungal and bacterial origin has received much attention in research during the past ten to fifteen years since their discovery (Agger et al. 2023; Biely 2016; Larsbrink and Lo Leggio 2023). Activity has been demonstrated widely on both synthetic and natural substrates, and several crystal structures reveal the catalytic mechanism of these canonical α/β-hydrolases of the serine-esterase type (Baath et al. 2019; Charavgi et al. 2013; Ernst et al. 2020; Mazurkewich et al. 2019; Topakas et al. 2010). We know that fungal variants are generally more restricted in their substrate preferences than the bacterial enzymes, because most of the fungal ones are dependent on the 4-*O*-methyl-modification on the glucuronoyl moiety for recognition. Bacterial enzymes are broader in their substrate preferences in terms of the sugar-moiety, but it is still uncertain for all types of glucuronoyl esterases, if they have specificity towards the configuration of the lignin-alcohol and if so, which of the two possible variants they prefer.

We have investigated the substrate specificity of a fungal glucuronoyl esterase from *Cerrena unicolor* (*Cu*GE) by exploiting the fact that esterases often display transesterification capacities under the right reaction conditions. We hypothesize that the enzyme will preferably perform transesterification with the type of alcohol (α- or γ-positioned) that fits its substrate specificities best. We performed transesterifications with either methyl-glucuronate or methyl-4-*O*-methyl-glucuronate as donor substrates, and benzyl-alcohol or 3-phenyl-1-propanol as the alcohol-acceptors according to the four reactions outlined in Fig. 1.

## Materials and enzyme preparation

Methyl 4-*O*-methyl-D-glucopyranosyluronate (Me-4-O-MeGlcA) was purchased from Institute of Chemistry, Slovak Academy of Sciences, Bratislava, Slovakia. Glucuronic acid methyl ester (Me-GlcA) and benzyl D-glucuronate (Bnz-GlcA) were purchased from Carbosynth. Benzyl alcohol (BnzOH), 3-phenyl-1-propanol (PhPrOH) and all other chemicals were purchased from Sigma.

The construct containing the gene encoding for the CE15 glucuronoyl esterase from *Cerrena unicolor* (*Cu*GE) was produced in *P. pastoris* as previously described (Mosbech et al. 2018). After fermentation the cells were separated by centrifugation and the fermentation broth (3 L) was sterile filtered and concentrated by ultrafiltration on a 10 kDa cut-off membrane to a final volume of approx. 80 mL. *Cu*GE was purified by affinity chromatography on an IMAC-column (HisTrap HP 5 mL column, GE Healthcare) using an Äkta Purifier 100 (GE Healthcare, Uppsala Sweden).

## Enzyme reactions

The transesterification activity of *Cu*GE was tested in four different experimental setups using either Me-4-*O*-MeGlcA or Me-GlcA as donor substrates and benzyl alcohol (BnzOH) or 3-phenyl-1-propanol (PhPrOH) as acceptor substrates, allowing the assessment of the transesterification ability and preferences of *Cu*GE in either the α and γ position (Fig. 1).

Transesterification with PhPrOH as acceptor molecule was performed in a 100 µL reaction mixture containing 0.45 mM of Me-4-*O*-MeGlcA or 0.48 mM of Me-GlcA (dissolved in 10 mM Na acetate buffer pH 6) and 6.32 M of PhPrOH in order to have an acceptor–donor ratio of approx. 14,000 and of 13,000 with Me-4-*O*-MeGlcA and Me-GlcA, respectively. The reaction was started in a HPLC vial by addition of 0.02 µM or 0.87 µM of *Cu*GE for the reaction with Me-4-*O*-MeGlcA and Me-GlcA, respectively.

Transesterification with BnzOH as acceptor molecule was performed in a 100 µL reaction mixture containing 0.45 mM of Me-4-*O*-MeGlcA or 0.48 mM of Me-GlcA (dissolved in 10 mM Na acetate buffer pH 6) and 8.31 M of BnzOH in order to have an acceptor donor ratio of approx.. 18,500 and of 17,500 with Me-4-*O*-MeGlcA and Me-GlcA, respectively. The reaction was started in a HPLC vial by addition of 0.11 µM or 4.33 µM of *Cu*GE for the reaction with Me-4-*O*-MeGlcA and Me-GlcA, respectively. All transesterification reactions were run in triplicates.

The reaction samples were placed in the UHPLC’s autosampler at 40 °C and the reaction evolution was followed directly on LC–MS by injecting 5 µL of the reaction mixture onto the column every 20 min followed by the LC–MS method described below.

Control experiments containing 0.45 mM of Me-4-*O*-MeGlcA or 0.48 mM of Me-GlcA (dissolved in 10 mM Na acetate buffer pH 6) and 6.32 M of PhPrOH or 8.31 M of BnzOH and replacing the enzyme volume with an equal amount of 10 mM Na acetate buffer pH 6 were run to assess auto-hydrolysis during the reaction time.

Chromatograms of all transesterification reactions and auto-hydrolytic control experiments are found in Supplementary Figs. S1-S8.

Control experiments showing *Cu*GE’s hydrolytic activity were performed in 100 µL reaction mixture containing 0.45 mM of Me-4-*O*-MeGlcA or 0.48 mM of Me-GlcA dissolved in 10 mM Na acetate buffer pH 6. The reactions were started in the HPLC vial by the addition of 0.08 µM or 4.33 µM of *Cu*GE for the reaction with Me-4-*O*-MeGlcA and Me-GlcA, respectively. The hydrolytic reactions were performed in triplicates. Direct quantification results of substrate depletion are provided in Supplementary Table S1.

## LC–MS method

Reaction product profiles were analyzed by LC–MS. 5 µL of reaction mixture was injected onto a Hypercarb column (150 mm × 2.1 mm; 3μm, Thermo Fischer Scientific, Sunnyvale, CA, USA). The chromatography was performed on a Dionex UltiMate 3000 UPLC (Thermo Fischer Scientific, Sunnyvale, CA, USA) at 0.4 mL min^−1^ and 70 °C with a two-eluent system consisting of eluent A (acetonitrile) and eluent B (water). The elution was performed as follows (time indicated in min): 0–3, 0% A 100% B; 3–10, isocratic 40% A 60% B; 10–19, isocratic 0% A 100% B. Water was used as eluent B to avoid excessive spontaneous hydrolysis of either donor substrates or products on the LC-column.

For the analysis of the transesterification of PhPrOH and Me-4-*O*-MeGlcA the chromatography was performed on the same system and condition of flow, temperature and eluent system but the elution was performed as follows (time indicated in min): 0–3, 0% A 100% B; 3–10, isocratic 70% A 30% B; 10–19, isocratic 0% A 100% B. The differences in elution system for this particular reaction was in order to elute the stronger retained transesterification product of this reaction compared to the other reactions within the same period.

The HPLC was connected to an ESI-iontrap (model Amazon SL from Bruker Daltonics, Bremen, Germany) and the electrospray was operated in positive ultra scan mode using a target mass of 300 m/z. A scan range from 100 to 2000 m/z was selected and capillary voltage was set to 4.5 kV, end plate offset 0.5 kV, nebulizer pressure at 3.0 bar, dry gas flow at 12.0 L min^−1^, and dry gas temperature at 280 °C.

## Quantification method

Quantification of all precursor ions was performed using Bruker TASQ software (Bruker Daltonics, Bremen, Germany). All ions were observed as [M + Na]^+^.

Quantification of the Me-4-*O*-MeGlcA hydrolysis by *Cu*GE was performed by defining an Extracted Ion Chromatogram (EIC) of *m/z* 244.95 and *m/z* 467.03 (pseudo-double ion with one sodium), with a width of ± 0.5 and retention time 6.5 min ± 0.2 min, and reaction extend was quantified as substrate depletion relative to calibration with Me-4-*O*-MeGlcA.

Quantification of the Me-GlcA hydrolysis by *Cu*GE was performed by defining an EIC of *m/z* 230.92 and *m/z* 439.07 (pseudo-double ion with one sodium), with a width of ± 0.5, retention time 3.0 min ± 0.2 min, and reaction extent was quantified as substrate depletion relative to calibration with Me-GlcA.

Quantification of product formations of both Bnz-4-*O*-MeGlcA (EIC on *m/z* 321.08 with a width of ± 0.5, retention time 9.8 min) and Bnz-GlcA (EIC on *m/z* 307.04 and *m/z* 591.01 with a width of ± 0.5, retention time 8.0 min), were performed relative to a Bnz-GlcA standard dissolved in BnzOH (EIC on *m/z* 307.04 and *m/z* 591.01 with a width of ± 0.5 and retention time 8.0 min with a window of 0.2 min).

Estimations of PhPr-4-*O*-MeGlcA (EIC on *m/z* 349.14, retention time 9.3 min) and PhPr-GlcA (EIC on *m/z* 335.12, retention time 7.7 min); products of *Cu*GE transesterification of PhPrOH with Me-4-*O*-MeGlcA or Me-GlcA, respectively, were performed using Bnz-GlcA diluted in PhPrOH as standard.

Calibration curves was performed using 6–8 levels of concentrations and fitting the data with a quadratic curve for the Me-4-*O*-MeGlcA and Me-GlcA calibrations and with a linear curve for the Bnz-GlcA (Supplementary Figure S9).

## Results and discussion

**Fig. 2:**
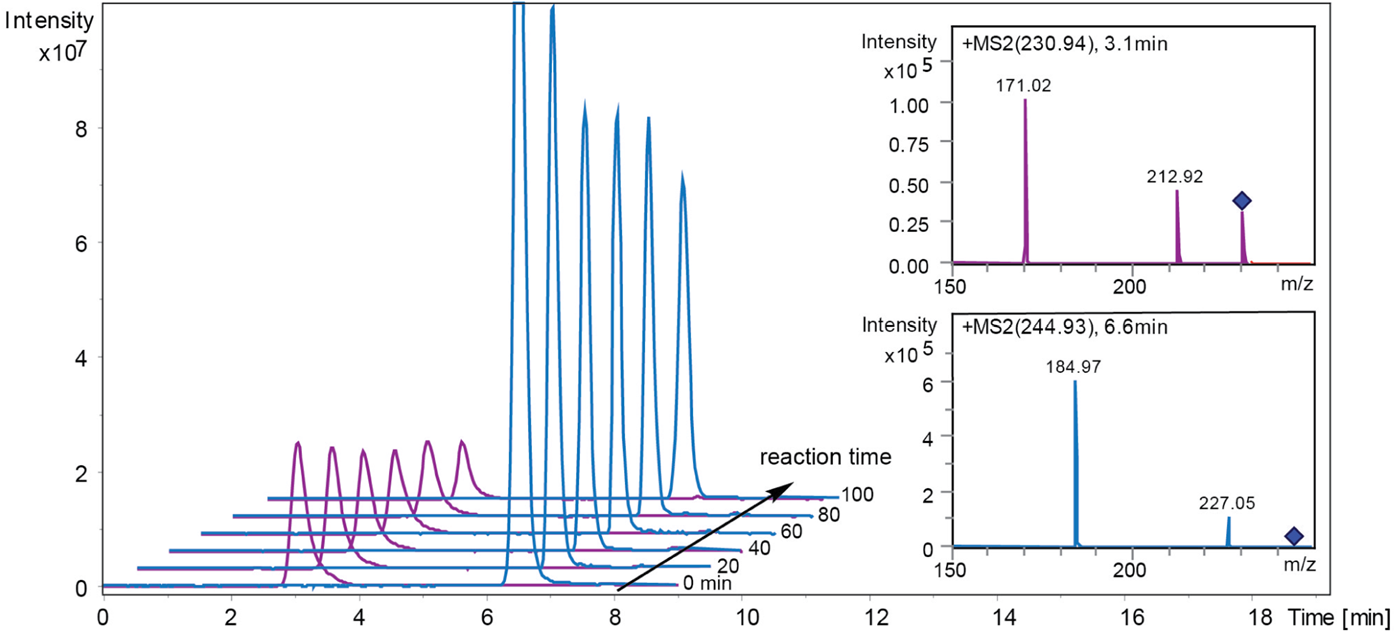
Comparison between the hydrolysis of Me-4-*O*-MeGlcA and Me-GlcA by *Cu*GE. Reaction evolution over 100 min of the Me-GlcA hydrolysis by (4.3 µM) *Cu*GE is showed in violet at retention time (RT) 3.0 min. MS2 spectrum of Me-GlcA m/z 230.92 is reported in the upper right corner (violet). Reaction evolution over 100 min of the Me-4-*O*-MeGlcA hydrolysis by (0.087 µM) *Cu*GE is showed in blue at RT 6.5 min. MS2 spectrum of Me-4-*O*-MeGlcA m/z 244.95 is reported in the lower right corner (blue). All ions are shown as [M + Na]^+^. The two hydrolysis were run separately

Initial hydrolytic experiments with either Me-4-*O*-MeGlcA or Me-GlcA as substrate and in the absence of alcohol acceptors show substrate conversion as expected (Fig. 2), with a significantly higher level of conversion for the 4-*O*-methylated ester compared to the non-derivatized glucuronate ester within the first 100 min of reaction (Table 1). Enzyme concentrations were dosed to allow similar rates of catalysis in all experiments, independent of substrate, and consequently *Cu*GE was dosed 50 times less in the experiments where Me-4-*O*-MeGlcA was the substrate compared to the experiments with Me-GlcA as substrate. The fact that *Cu*GE prefers its substrates to have the 4-*O*-methyl-modification on the glucuronoyl is well-establised (d’Errico et al. 2015; Ernst et al. 2020; Monrad et al. 2018). As the 100 min reaction time progresses, it becomes evident that the hydrolytic reaction slows down for both substrates (Table 1), which again is not surprising as the affinity (K_M_) for these synthetic model substrates is known to be relatively high (d’Errico et al. 2015).

**Table 1:** Comparison of substrate conversion of either Me-4-*O*-MeGlcA or Me-GlcA relative to enzyme dosage within 100 min reaction time

Reaction time	Accumulated substrate conversion		Substrate conversion within each time interval	
	Me-4-*O*-MeGlcA	Me-GlcA	Me-4-*O*-MeGlcA	Me-GlcA
min	mmol/µmol *Cu*GE	mmol/µmol *Cu*GE	mmol/µmol *Cu*GE	mmol/µmol *Cu*GE
20	2.3 ± 0.1	0.05 ± 0.00	2.31 ± 0.1	0.05 ± 0.00
40	3.0 ± 0.08	0.07 ± 0.00	0.91 ± 0.03	0.02 ± 0.00
60	3.2 ± 0.03	0.08 ± 0.00	0.91 ± 0.05	0.02 ± 0.01
80	3.6 ± 0.13	0.09 ± 0.01	0.52 ± 0.00	0.02 ± 0.01
100	3.6 ± 0.1	0.10 ± 0.01	0.43 ± 0.12	0.01 ± 0.00

Accumulated substrate conversion signifies how much substrate is converted at a given time point relative to the enzyme concentration. Substrate conversion within each time interval describes how much additional substrate is converted at a given time point since the previous, relative to the enzyme concentration

**Fig. 3:**
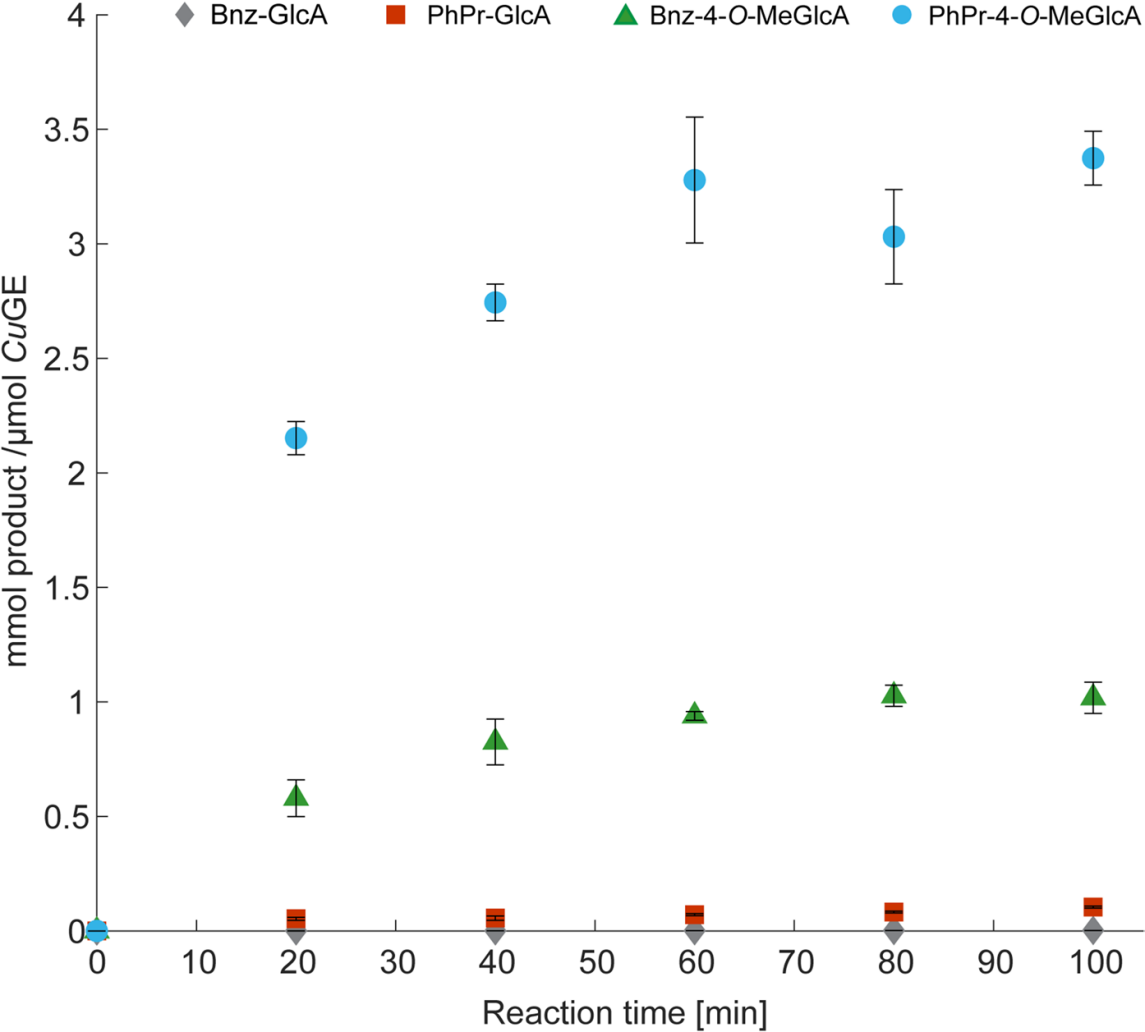
Product formation after transesterification reactions catalyzed by *Cu*GE. Reactions are combinations of Me-GlcA or Me-4-*O*-MeGlcA with either benzyl-alcohol or 3-phenyl-1-propanol according to reactions in Fig. 1. Blue circle: PhPr-4-*O*-MeGlcA ester (product reaction 1, Fig. 1). *Cu*GE dosage; 0.02 µM. Green triangle: Bnz-4-*O*-MeGlcA ester (product reaction 2, Fig. 1). *Cu*GE dosage; 0.11 µM. Red square: PhPr-GlcA ester (product reaction 3, Fig. 1). *Cu*GE dosage; 0.87 µM. Grey diamond: Bnz-GlcA ester (product reaction 4, Fig. 1). *Cu*GE dosage; 4.3 µM. Individual plots of each reaction is found in Supplementary Figure S10

It is known, that glucuronoyl esterases prefer bulky alcohols and in that respect, the methyl-esters are poor substrates. Experiments with either benzyl-alcohol or 3-phenyl-1-propanol as the primary solvent and thereby dominating acceptor molecule (14% v/v water), show that the transesterification reaction occurring between Me-4-*O*-MeGlcA and PhPrOH (Fig. 1, reaction 1) is significantly higher than any of the other combinations (Fig. 3). The reaction between Me-4-*O*-MeGlcA and BnzOH is second best (Fig. 1, reaction 2) with about 3–4 fold less product compared to the PhPr-GlcA ester. Quantifications of the ester products are done relative to the standard of Bnz-GlcA dissolved either in BnzOH or PhPrOH depending on the relevant acceptor alcohol, and the calibrations indicate a certain level of ion suppression when PhPrOH is the solvent (Supplementary standards curves), hence leading to a potential underestimation in that respect. PhPrOH has similar retention time as the transesterification products. At the same time, compounds carrying the 4-*O*-methylation tends to ionize better, and therefore give stronger responses. In the reactions performed here, enzyme loadings vary according to formation of transesterification products, as the goal is to quantify transesterification products. Hence, at low enzyme loadings, hydrolysis is not observed for reactions containing Me-4-*O*-MeGlcA as donor (and relatively low water concentration), whereas increasing the enzyme concentration yields hydrolytic reactions in parallel to the transesterification (data not shown). Ultimately, the extent of hydrolysis is a competition for acceptor substrate, which appears favored in the case of the alternative alcohol when enzyme concentrations are low. Hydrolytic reactions are certainly present but becomes un-quantifiable at conditions where transesterification is dominating.

**Fig. 4:**
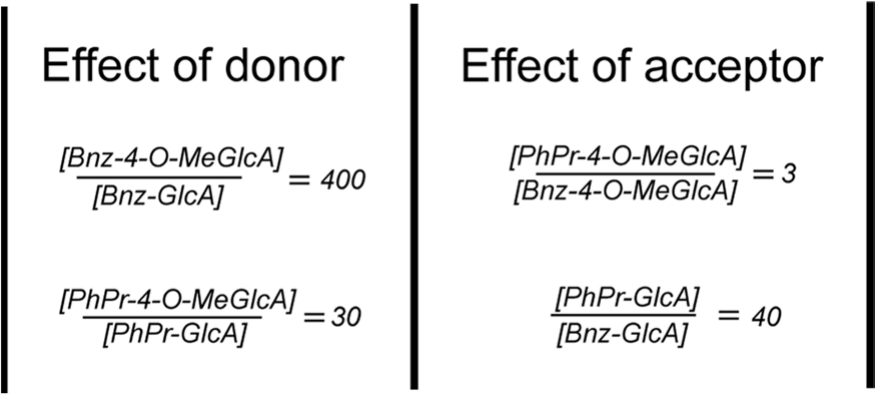
Ratios between concentrations of product formations, which illustrates the effect of changing the donor (left hand side), or changing the acceptor (right hand side). The ratios are calculated based on product concentrations at 100 min reaction time. On the left hand side, the donor substrate is changed, and the acceptor is kept constant. In the right hand side, the acceptor is changed and the donor is kept constant

Interestingly, the 4-*O*-methylation is a more important feature for catalysis than the nature of the acceptor-alcohol, since the Bnz-4-*O*-MeGlcA-ester forms faster than the PhPr-GlcA ester. A cautious estimation of the effect of the donor versus the effect of the acceptor demonstrates that the ratio between concentrations of products formed during the reaction time is higher when the donor is changed, compared to when the acceptor is changed (Fig. 4).

Hence, the effect of changing the donor substrate between the 4-*O*-methylated and non-methylated counterparts is larger than the effect of changing the alcohol acceptor. This observation may not be valid for all glucuronoyl esterases, as it is common knowledge that not all GEs favor the 4-*O*-methylation.

Recent QM/MM studies of the fungal *Thermothelomyces thermophila* glucuronoyl esterase (*Tt*GE) show that the acylation step is the most energy demanding and hence rate-limiting step (Viegas et al. 2022), whereas similar studies with a bacterial *Ot*CE15A from *Opitutus terrae* show that the deacylation-step is rate-limiting (Zong et al. 2022). Without having investigated the energetic landscape of catalysis by *Cu*GE, the transesterification reactions we observe support the observation that the acylation-step is determining for reaction speed. However, it is well-known that the differences between fungal and bacterial GEs are quite large in terms of overall structure (Larsbrink and Lo Leggio 2023), and transesterification reactions of the kind presented here, may turn out different for bacterial GEs with respect to energetic fingerprints.

The aim of this study is to investigate the preference for either an α- or a γ-positioned alcohol, and the results show that the γ-positioned alcohol is most favorable, yet the enzyme can perform the reaction with the α-alcohol. There are currently no univocal structural explanations for how the GEs interact with the alcohol moiety of the substrate. However, we have recently performed docking simulations with these exact two esters; Bnz-4-*O*-MeGlcA and PhPr-4-*O*-MeGlcA as ligands in *Cu*GE and guided by the sugar-moiety, which show steric obstacles when the α-benzyl constitutes the alcohol part of the ligand in contrary to the γ-linked ester (Agger et al. 2023).

Exploiting the transesterification capacities of these enzymes potentially opens for other biotechnological applications than biomass deconstruction, such as functionalization of hemicellulose or smaller aldouronic acids with alcohols of different properties (hydrophobicity, other functional groups etc.). Future studies may explore the field of potential acceptors more broadly than here, and thereby determine the diversity of alcohols and properties that could be relevant to investigate further.

## Conclusion

*Cu*GE prefers methyl-4-*O*-methyl-glucuronate as donor substrate and the γ-positioned acceptor according to the experiments conducted here, and evaluated based on yield and rate of product formation. It is important to emphasize that the results reported here illustrate the substrate preferences of *Cu*GE, which is not to be generalized for all GEs. These results exemplify a methodology for investigating substrate preferences, and future studies may include reverse hydrolytic reactions.

We observe large differences in product formation rates during the first 100 min of reaction with the formation of the 4-*O*-methyl-glucuronoyl-3-propane-phenyl as fastest, and the results clearly indicate that *Cu*GE prefers the γ-positioned alcohol during transesterification. The most important factor for reaction though, continues to be the presence of the 4-*O*-methylation derivatization of the glucuronoyl moiety.

These experiments demonstrate a methodology where the enzyme reveals its substrate preferences immediately by favoring product formation from the most suitable substrates. These results do not inform about the prevalence of either α- or γ-LCC esters in lignocellulosic biomass, but it certainly informs about the preferences of this particular enzyme. Given that enzymes evolve to tackle the linkages present in biomass, it is tempting to speculate that the γ-esters LCC is the more prevalent of the two types in biomass.

### Supplementary Information

Below is the link to the electronic supplementary material.

Supplementary file1 (PDF 3147 KB)
