# Supplementary material for: Transesterification with CE15 glucuronoyl esterase from Cerrena unicolor reveals substrate preferences
Source: Biotechnol Lett. 2023 Dec 27;46(1):107–14. doi: 10.1007/s10529-023-03456-x (PMC10787888; doi:10.1007/s10529-023-03456-x)
Supplement: Supplementary file 1 — Supplementary file1 (PDF 3147 KB) [file 10529_2023_3456_MOESM1_ESM.pdf]

Supplementary information for

Transesterification with CE15 glucuronoyl esterase from *Cerrena unicolor* reveals substrate preferences

Biotechnology Letters

*Authors: Valentina Perna and Jane Wittrup Agger**

Department of Biotechnology and Biomedicine

Technical University of Denmark

2800 Kgs Lyngby, Denmark

*corresponding; jaag@dtu.dk

Table S1. **Results of control experiments of Me-4-*O*-MeGlcA and Me-GlcA hydrolysis by *Cu*GE**. Me-4-*O*-MeGlcA hydrolysis was performed with 5.2 µg/mL of CuGE while Me-GlcA hydrolysis was performed with 260 µg/mL of CuGE.

Reaction time	Me-4-*O*-MeGlcA	MeGlcA
	[mM]	[mM]
0	0.49 ± 0.09	0.53 ± 0.01
20	0.41 ± 0.12	0.38 ± 0.01
40	0.33 ± 0.09	0.31 ± 0.03
60	0.29 ± 0.06	0.23 ± 0.09
80	0.24 ± 0.04	0.17 ± 0.06
90	0.22 ± 0.01	0.09 ± 0.03

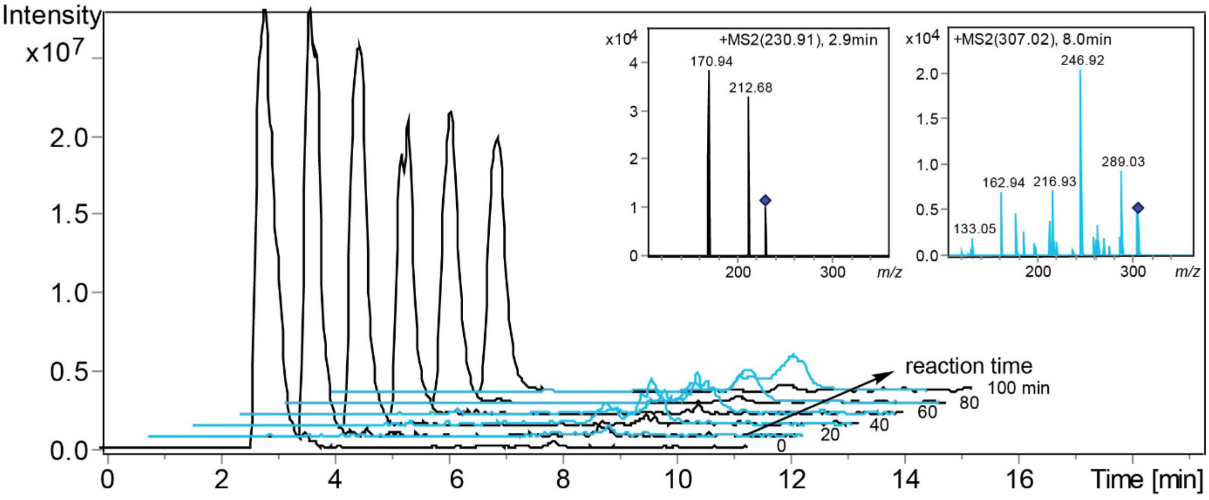

Figure S1. Reaction progression of the transesterification of BnzOH with Me-GlcA by 260 µg/mL (4.3 µM) *Cu*GE over 100 min. Extracted Ion Chromatogram (EIC) of Me-GlcA (black) at a retention time (RT) 2.9 min and *m/z* 230.91 decreased intensity over reaction time. EIC of BnzGlcA (blue) at a RT 8.0 min and *m/z* 307.02 increased intensity over reaction time. All ions are shown as $[M+Na]^+$. MS2 spectra of *m/z* 230.91 and *m/z* 307.02 are also shown.

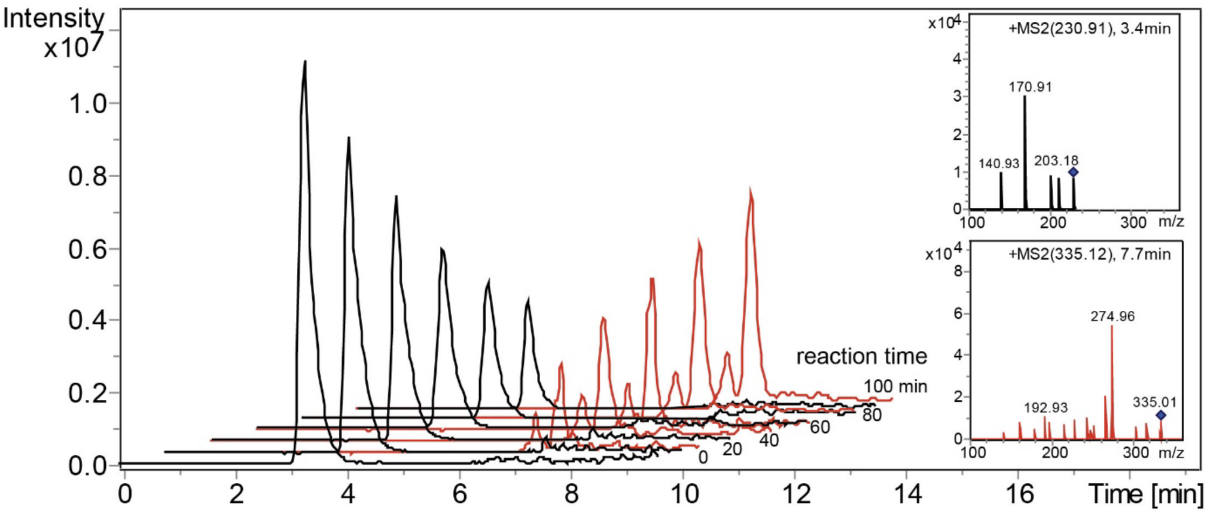

Figure S2. Reaction progression of the transesterification of PhPrOH with Me-GlcA by 52 µg/mL (0.87 µM) *Cu*GE over 100 min. EIC of Me-GlcA (black) at a retention time (RT) 3.4 min and *m/z* 230.91 decreased intensity over reaction time. EIC of PhPr-GlcA (red) at a RT 7.7 min and *m/z* 335.12 increased intensity over reaction time. All ions are shown as $[M+Na]^+$. MS2 spectra of *m/z* 230.91 and *m/z* 335.12 are also shown. *m/z* 335.12 splits in two peaks (a minor shoulder in front of the main peak), which could be indicative for chirality, however not investigated further.

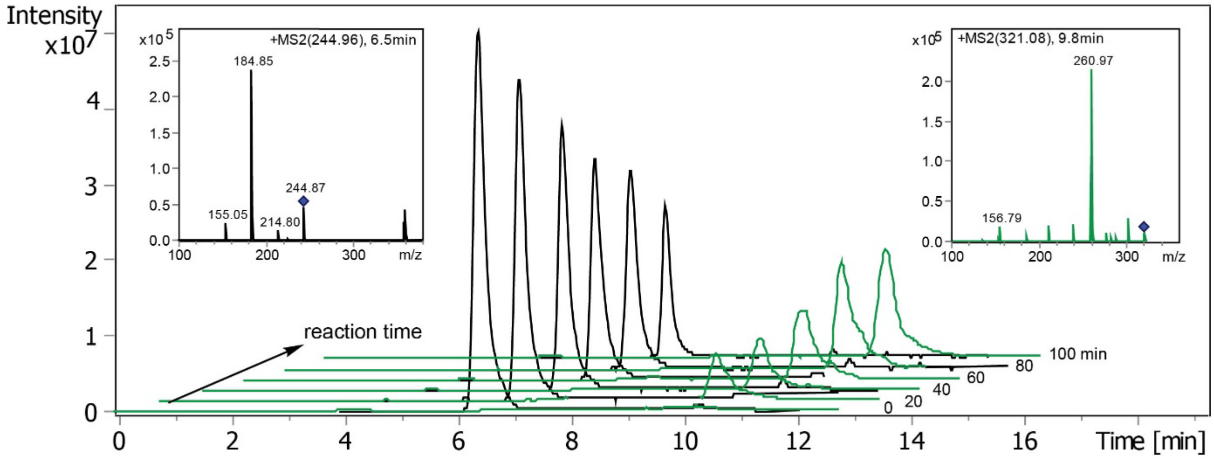

Figure S3. Reaction progression of the transesterification of BnzOH with Me-4-*O*-MeGlcA by 6.5 µg/mL (0.11 µM) *Cu*GE over 100 min. EIC of Me-4-*O*-MeGlcA (black) at a retention time (RT) 6.5 min and *m/z* 244.96 decreased intensity over reaction time. Bnz-4-*O*-MeGlcA (green) at a RT 9.8 min and *m/z* 321.08 increased intensity over reaction time. All ions are shown as $[M+Na]^+$. MS2 spectra of *m/z* 244.96 and *m/z* 321.08 are also shown.

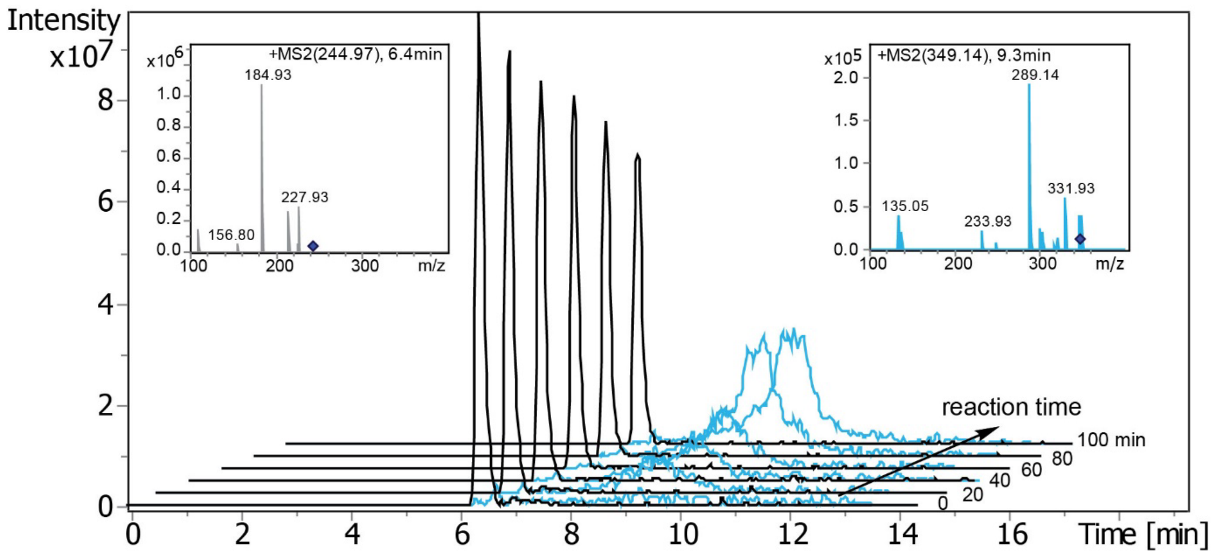

Figure S4. Reaction progression of the transesterification of PhPrOH with Me-4-*O*-MeGlcA by 1.3 µg/mL (0.02 µM) *Cu*GE over 100 min. EIC of Me-4-*O*-MeGlcA (black) at a retention time (RT) 6.4 min and *m/z* 244.97 decreased intensity over reaction time. PhPr-4-*O*-MeGlcA (blue) at a RT 9.3 min and *m/z* 349.14 increased intensity over reaction time. All ions are shown as $[M+Na]^+$. MS2 spectra of *m/z* 244.97 and *m/z* 349.14 are also shown.

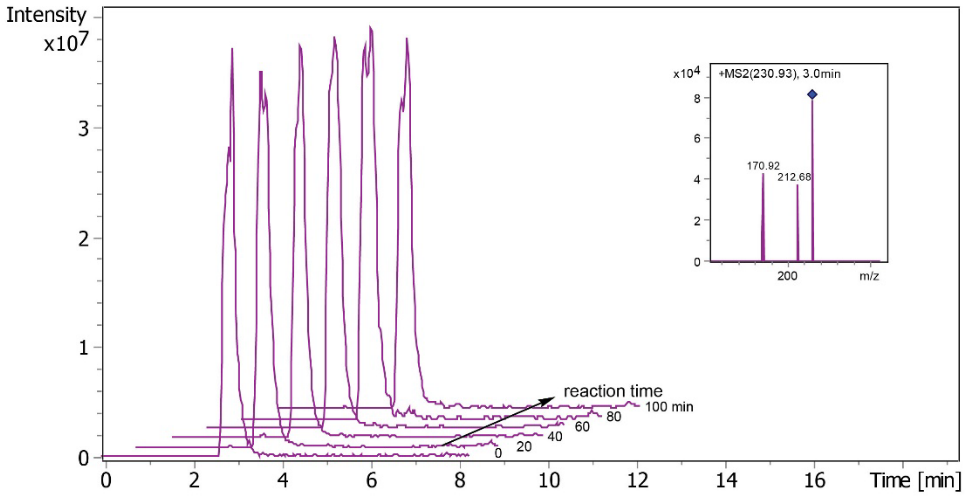

Figure S5. Control reaction with BnzOH and Me-GlcA where *Cu*GE loading was substituted with 10 mM Na acetate buffer pH 6 over 100 min. Extracted Ion Chromatogram (EIC) of Me-GlcA (violet) at a retention time (RT) 3.0 min and *m/z* 230.91 remained constant in intensity over reaction time. Ion is shown as $[M+Na]^+$. MS2 spectrum of *m/z* 230.91 is also shown.

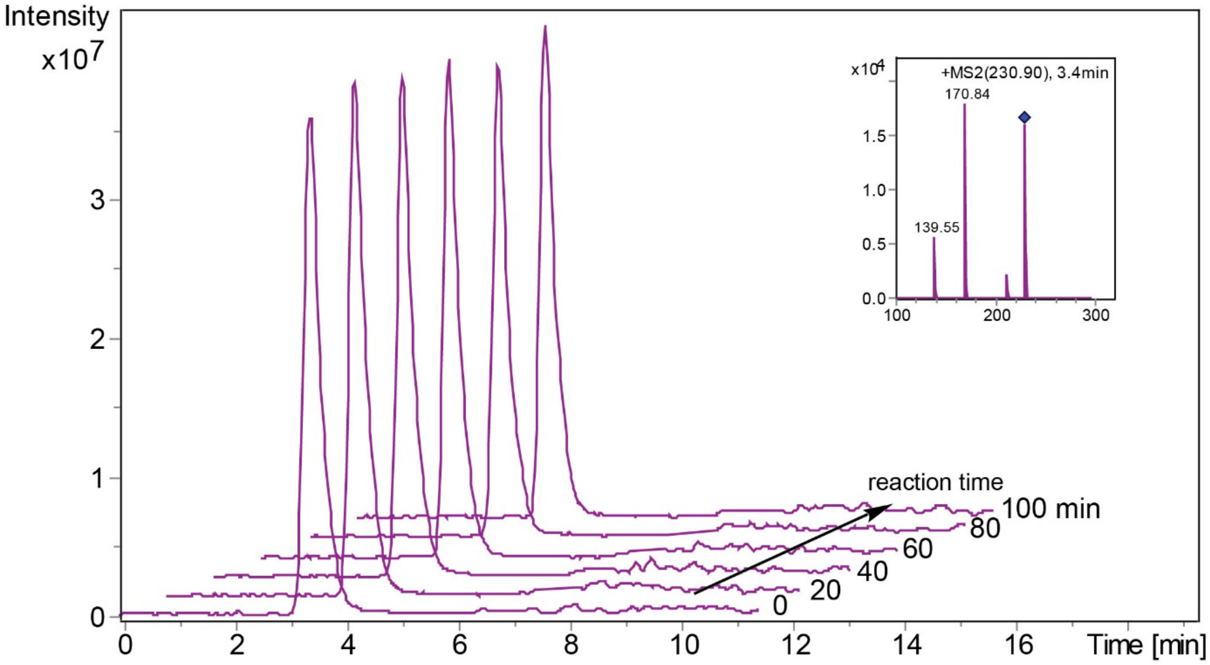

Figure S6. Control reaction with PhPrOH and Me-GlcA where *Cu*GE loading was substituted with 10 mM Na acetate buffer pH 6 over 100 min. Extracted Ion Chromatogram (EIC) of Me-GlcA (violet) at a retention time (RT) 3.4 min and *m/z* 230.90 remained constant in intensity over reaction time. Ion is shown as $[M+Na]^+$. MS2 spectrum of *m/z* 230.90 is also shown.

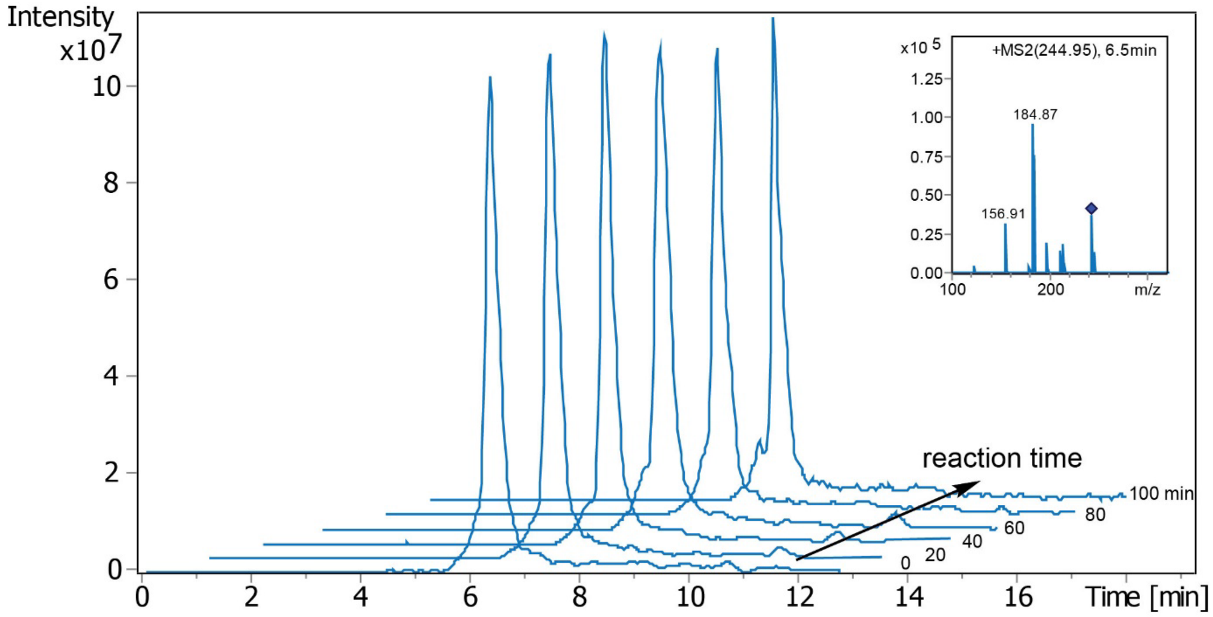

Figure S7. Control reaction with BnzOH and Me-4-*O*-MeGlcA where *Cu*GE loading was substituted with 10 mM Na acetate buffer pH 6 over 100 min. Extracted Ion Chromatogram (EIC) of Me-4-*O*-MeGlcA (blue) at a retention time (RT) 6.5 min and *m/z* 244.95 remained constant in intensity over reaction time. Ion is shown as $[M+Na]^{+}$. MS2 spectrum of *m/z* 244.95 is also shown.

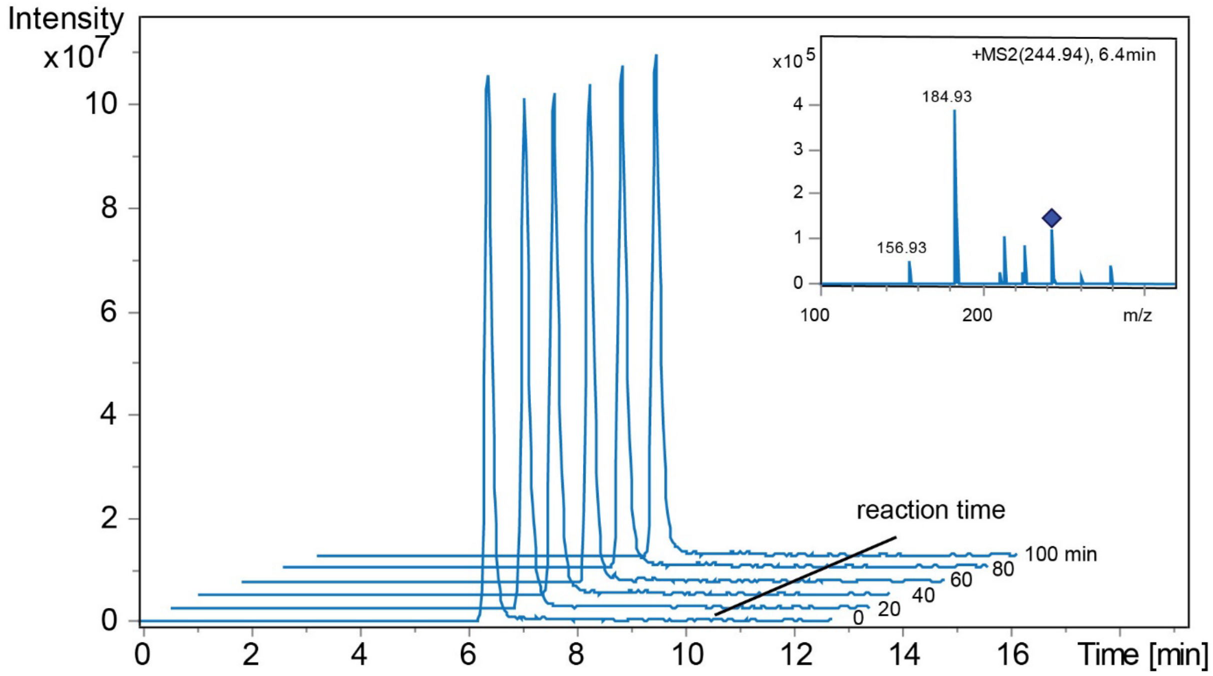

Figure S8. Control reaction with PhPrOH and Me-4-*O*-MeGlcA where *Cu*GE loading was substituted with 10 mM Na acetate buffer pH 6 over 100 min. Extracted Ion Chromatogram (EIC) of Me-4-*O*-MeGlcA (blue) at a retention time (RT) 6.4 min and *m/z* 244.94 remained constant in intensity over reaction time. Ion is shown as $[M+Na]^{+}$. MS2 spectrum of *m/z* 244.95 is also shown.

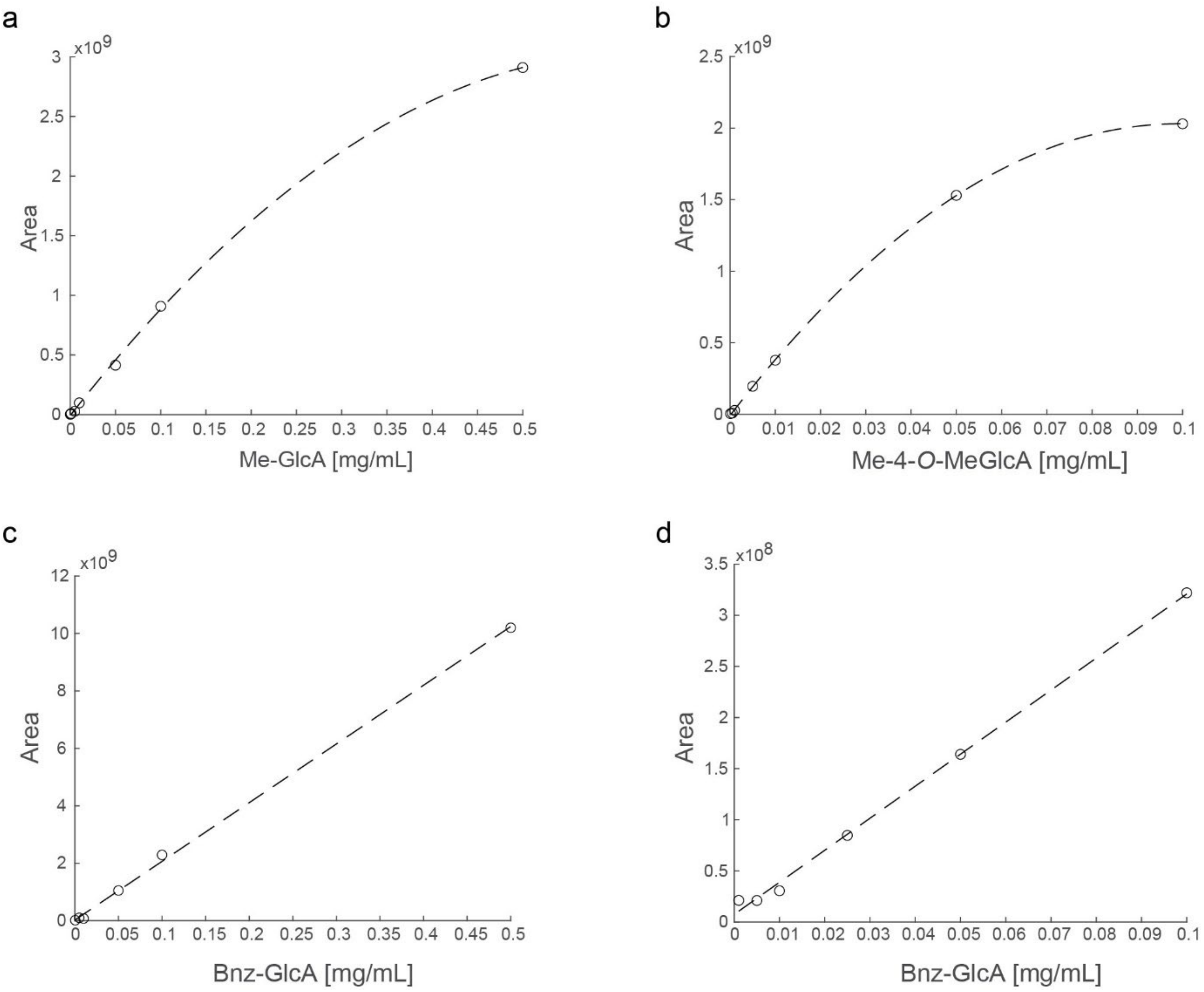

Figure S9. Calibration curves. a) Calibration curve used for Me-GlcA hydrolysis by *Cu*GE determined as the area below the EIC of *m/z* 230.92 and *m/z* 439.07 at different MeGlcA concentrations. b) Calibration curve used for Me-4-*O*-MeGlcA hydrolysis by *Cu*GE determined as the area below the EIC of *m/z* 244.95 and *m/z* 467.03 at different Me-4-*O*-MeGlcA concentrations. c) Calibration curve used for transesterification products of BnzOH with Me-4-*O*-MeGlcA and Me-GlcA. Quantification is performed relative to the concentration of BnzGlcA (dissolved in BnzOH, as the area below the EIC of *m/z* 307.04 and *m/z* 591.01). d) Calibration curve used for transesterification of PhPrOH with Me-4-*O*-MeGlcA and Me-GlcA. Quantification is performed relative to the concentration of Bnz-GlcA (dissolved in PhPrOH, area below the EIC of *m/z* 307.04 and *m/z* 591.01). All calibrations are performed in triplicate.

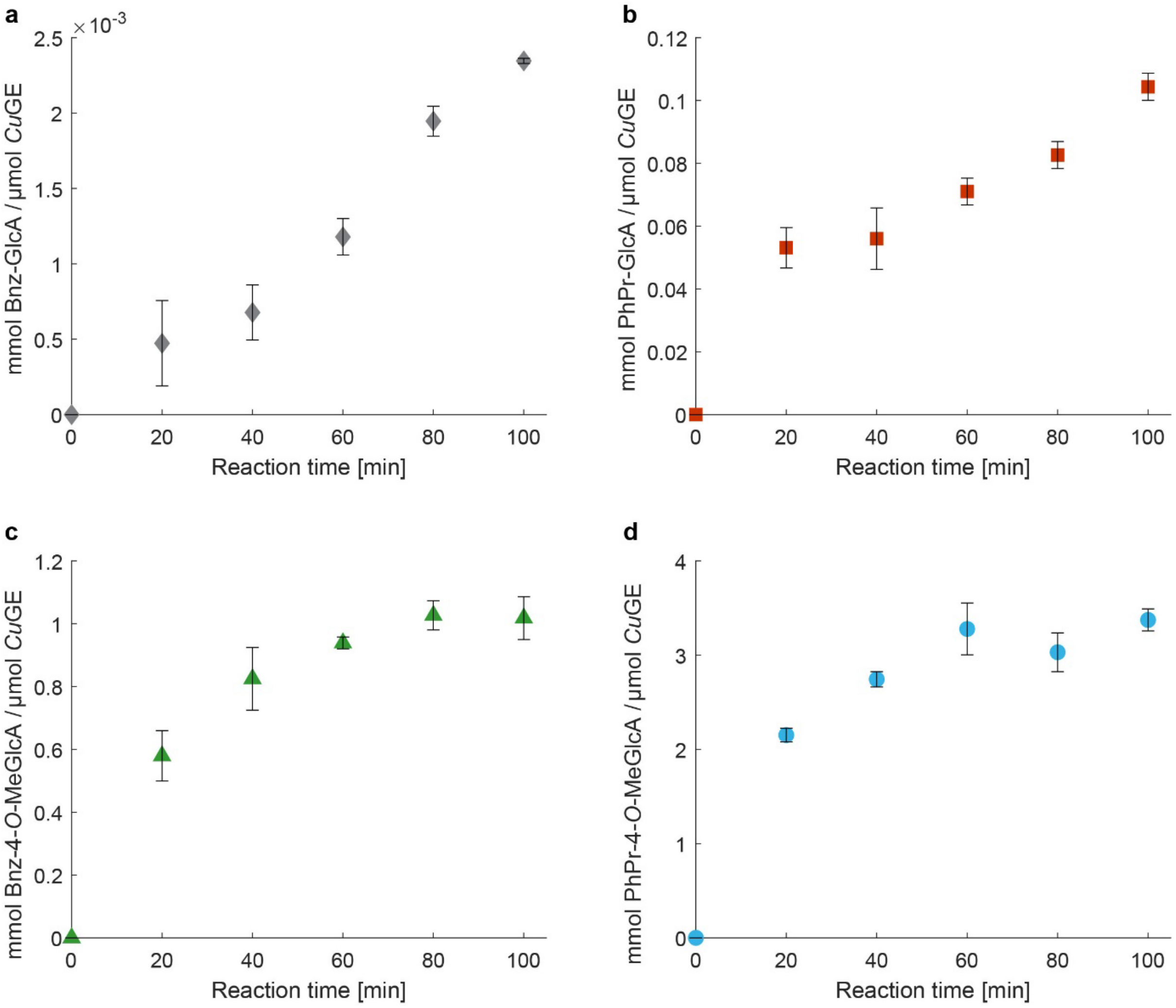

Figure S10. Comparison between the product formations during transesterification reactions by *Cu*GE. a) Product formation of the ester-product Bnz-GlcA (grey diamonds) from the transesterification of BnzOH with Me-GlcA expressed as product formation (mmol Bnz-GlcA/µmol *Cu*GE) using 260 µg/mL (4.3 µM) of *Cu*GE. b) Product formation of the ester-product PhPr-GlcA (red squares) from the transesterification of PhPrOH with Me-GlcA using 52 µg/mL (0.87 µM) of *Cu*GE. c) Product formation of the ester-product Bnz-4-*O*-MeGlcA (grey diamonds) from the transesterification of BnzOH with Me-4-*O*-MeGlcA using 6.5 µg/mL (0.11 µM) of *Cu*GE. d) Product formation of the ester-product PhPr-4-*O*-MeGlcA (light blue circles) from the transesterification of PhPrOH with Me-4-*O*-MeGlcA using 1.3 µg/mL (0.02 µM) of *Cu*GE. Standard deviations of triplicate reactions are shown. The reaction evolution cannot be compared directly, as they are showed with their respective y-axis.
